# Nutritional Inadequacies and Body Composition in Czech Competitive Rhythmic Gymnasts: A Pilot Study

**DOI:** 10.3390/nu18213599

**Published:** 2026-10-30

**Authors:** Anna Jílková, Kateřina Savková, Diana Chrpová, Lenka Kouřimská

**Affiliations:** Faculty of Agrobiology, Food and Natural Resources, Czech University of Life Sciences Prague, 165 00 Praha, Czech Republic; savkakat@seznam.cz (K.S.); chrpovad@af.czu.cz (D.C.); kourimska@af.czu.cz (L.K.)

**Keywords:** rhythmic gymnastics, dietary adequacy, female athletes, low energy availability (LEA), relative energy deficiency in sport (RED-S), body composition assessment, nutrition education

## Abstract

**Background/Objectives**: Rhythmic gymnastics combines high training demands with aesthetic pressure. This pilot study assessed dietary intake, body composition, and nutrition-related behaviours in Czech competitive rhythmic gymnasts. **Methods**: Fourteen female athletes (juniors aged 13–15 years, *n* = 6; seniors aged 16–20 years, *n* = 8) completed a three-day weighed food record at the baseline and underwent multifrequency bioelectrical impedance assessment at the baseline and approximately three months later, following individualised nutrition advice. A separate online questionnaire was completed by 50 gymnasts. **Results**: Reported energy intake averaged 63.7% and 67.1% of the estimated requirements among juniors and seniors, respectively. Carbohydrate, fibre, vitamin D, calcium, magnesium, and potassium intakes were low relative to prespecified study benchmarks. No statistically significant within-group changes in body composition were observed. An exploratory between-group difference in changes in the extracellular-to-total body water ratio was found (unadjusted *p* = 0.015), requiring cautious interpretation. Among the questionnaire respondents, 68% reported weight-related pressure, 60% intentional food restriction, and 56% had never heard of relative energy deficiency in sport (RED-S). **Conclusions**: Low reported intakes and weight-related concerns suggest potential need for qualified nutritional support. Intakes below the prespecified benchmarks do not establish individual nutritional inadequacy. The small uncontrolled cohort and absence of follow-up dietary records preclude conclusions about the effectiveness of nutritional counselling, while the short dietary assessment and age-independent benchmarks limit individual dietary interpretation and do not permit the diagnosis of clinical nutrient deficiencies, low energy availability, or RED-S.

## 1. Introduction

Rhythmic gymnastics is a physically demanding aesthetic sport requiring a combination of flexibility, strength, coordination, balance, technical precision, and repeated high-intensity training [1,2]. Meeting these demands requires adequate nutritional support to provide energy and nutrients for training, recovery, growth, and long-term athletic development. Contemporary sports nutrition recommendations therefore emphasise sufficient total energy intake, adequate carbohydrate availability, appropriate protein intake, micronutrient sufficiency, hydration, and suitable nutrient timing [3]. Nutritional strategies also play an important role in post-exercise recovery and the ability to maintain performance during subsequent training sessions [4]. Consequently, matching dietary intake to individual training demands is an important component of both performance-oriented and health-oriented support for athletes.

In rhythmic gymnastics, however, these nutritional requirements coexist with substantial aesthetic demands. A lean physique is traditionally associated with the sport, and athletes may experience pressure to maintain or reduce body mass despite high training loads. Body-image concerns and dissatisfaction have been repeatedly reported among rhythmic gymnasts [5], while participation in aesthetic sports has been associated with restrictive eating and disordered eating behaviours [6]. This creates a potential conflict between attempts to achieve the desired physique and the nutritional requirements needed to support training and physiological function. When energy intake is insufficient relative to exercise energy expenditure, athletes may develop low energy availability (LEA), which represents the underlying mechanism of relative energy deficiency in sport (RED-S). RED-S can adversely affect endocrine and reproductive function, bone health, immunity, gastrointestinal and cardiovascular function, psychological health, recovery, and athletic performance [7]. These consequences are particularly relevant to adolescent athletes, in whom adequate energy and nutrient availability must support both training and normal growth and maturation.

Body composition is an important consideration in many sports, but the relationship between leanness, health, and performance should not be oversimplified. Although lower body mass or body fat may be perceived as advantageous in aesthetic disciplines, there is no universally applicable “ideal” body composition for athletes, and aggressive weight-control practices may increase health and performance risks. Current best-practice recommendations therefore emphasise individualised assessment and caution against body-composition targets that are disconnected from an athlete’s health, developmental stage, nutritional status, and sporting requirements [8]. This is especially important in rhythmic gymnastics, where considerable variation exists between junior and senior athletes in terms of maturation, training volume, and body composition.

Previous studies indicate that dietary intake frequently fails to match these demands. Silva and Paiva (2014) reported low energy availability among female gymnasts before international competition [9], while Miteva et al. (2019) described insufficient energy intake and restrictive dietary practices in elite Bulgarian rhythmic gymnasts [10]. Similarly, Villa et al. identified inadequate dietary intake and a risk of LEA in elite competitive rhythmic gymnasts [11]. Insufficient carbohydrate intake is of particular relevance because carbohydrate availability contributes to the ability to sustain repeated high-intensity exercise and replenish glycogen stores between training sessions [3]. Micronutrient inadequacies have also been reported. Calcium and iron intake, for example, may be insufficient in adolescent gymnasts [12], which is important given the roles of these nutrients in bone development, oxygen transport, and physiological function. Collectively, these findings suggest that sports-nutrition support in rhythmic gymnastics should extend beyond body-mass management and address overall dietary adequacy, energy availability, recovery needs, and long-term athlete health.

An individualised approach may be particularly appropriate because nutritional requirements vary according to age, growth and maturation, training load, body composition, dietary habits, and individual goals. Furthermore, nutritional knowledge cannot be assumed even among competitive athletes. Recent research among adolescent gymnasts has demonstrated limited awareness of energy deficiency and its potential consequences [13]. Nutrition education interventions targeting LEA have shown potential to improve nutrition knowledge and dietary behaviours, although the available evidence remains heterogeneous and relatively limited [14]. Athletes also report a preference for practical nutrition education that includes reliable information, individual feedback, and access to qualified professionals [15]. Thus, combining the assessment of habitual dietary intake and body composition with individualised nutrition recommendations may provide a practical strategy for identifying athlete-specific inadequacies and translating sports-nutrition evidence into everyday practice. However, evidence regarding whether relatively short, individualised interventions result in measurable changes in body composition remains limited.

Although dietary practices and body composition have been examined in rhythmic gymnasts internationally, evidence is limited on Czech competitive athletes and on the joint presentation of baseline dietary records, two-point body-composition follow-up after individualised nutrition advice, and a separate national questionnaire addressing weight-related practices and RED-S awareness. The contribution of this study is therefore the descriptive integration of these complementary perspectives rather than proof of an intervention effect or a new diagnostic approach. The study was designed as a pilot because recruitment through competitive clubs yielded a small, non-random cohort suitable for exploratory assessment but insufficient for confirmatory age-group or intervention-effect inference. We aimed to describe dietary intake relative to prespecified protocol benchmarks and body-composition measurements at two visits in junior and senior gymnasts; a secondary aim was to describe nutrition-related behaviours in the questionnaire sample. Any between-group and longitudinal comparisons were exploratory.

## 2. Materials and Methods

### 2.1. Study Design and Ethical Approval

The study consisted of two complementary components: (1) a single-group nutrition intervention with repeated body-composition assessment in Czech competitive rhythmic gymnasts and (2) a nationwide online questionnaire survey designed to provide broader information on nutrition-related behaviours, weight-control practices, recovery, and nutrition knowledge among Czech competitive rhythmic gymnasts.

The intervention component included the baseline assessment of dietary intake and body composition, followed by individualised nutrition education and a second body-composition assessment approximately three months later.

The study was conducted in accordance with the Declaration of Helsinki and was approved by the Ethics Committee of the Czech University of Life Sciences Prague (Decision No. 01/2024, approved on 18 April 2024). Participation was voluntary. Written informed consent was obtained from all adult participants, and written informed consent for participants younger than 18 years was obtained from their legal representatives.

Generative artificial intelligence (GenAI, GPT-5.6 Sol; OpenAI, San Francisco, CA, USA) was used during manuscript preparation to support English translation, linguistic and structural refinement of selected sections, and the preparation of summary graphical visualisations based exclusively on the authors’ original data. AI tools were not used for study design, data collection, statistical analysis, or the generation of original research data. All AI-assisted outputs were critically reviewed, verified, and edited by the authors, who take full responsibility for the scientific content of the manuscript.

### 2.2. Study Component 1: Intervention Cohort—Participants and Recruitment

The intervention cohort comprised 14 female competitive rhythmic gymnasts. Participants were recruited by e-mail through selected rhythmic gymnastics clubs. Several clubs were approached; however, because recruitment was mediated by clubs, the exact number of athletes invited is unknown. Fourteen athletes consented and met the eligibility criteria. Three participating clubs were located in Prague and one in Ústí nad Labem, Czech Republic; measurements were conducted in Prague. No a priori sample-size or statistical-power calculation was undertaken. This was a small, pragmatic pilot cohort obtained through voluntary recruitment; accordingly, hypothesis tests are descriptive and exploratory rather than confirmatory.

Participants were divided according to competitive age category into juniors aged 13–15 years (*n* = 6) and seniors aged 16–20 years (*n* = 8). The inclusion criteria were female sex, age ≥ 13 years, active participation in competitive rhythmic gymnastics, regular membership in a Czech rhythmic gymnastics club, and absence of a serious acute or chronic condition likely to affect body composition or eating behaviour.

Exclusion criteria were pregnancy, a serious injury preventing regular training, prolonged illness during the follow-up period, discontinuation of competitive rhythmic gymnastics during the study, failure to complete the body-composition measurements, or failure to provide the required three-day food record.

The intervention cohort underwent dietary assessment and body-composition measurement at the baseline. Following the baseline assessment, athletes received nutrition education and individualised dietary recommendations. Body composition was reassessed approximately three months later.

### 2.3. Dietary Assessment

Short-term reported dietary intake was assessed using a three-day weighed food record consisting of two weekdays and one weekend day. Participants were instructed to weigh all foods and beverages consumed using household kitchen scales and to record the corresponding amounts as accurately as possible. For composite dishes, individual ingredients and their quantities were recorded whenever feasible. For each athlete, dietary intake was entered into Nutriservis Professional software (NutriServis, Prague, Czech Republic; https://www.nutriservis.cz/), and nutrient intake was calculated for each recorded day. According to the software provider, the food-composition database incorporates the Bratislava food-composition database and the Souci–Fachmann–Kraut tables, together with additional food and product entries. The specific software/database version used at the time of dietary processing and the source of each individual product entry could not be retrospectively verified. Mean daily intake across the three days was subsequently calculated for all variables included in the analysis. For the intervention cohort, dietary intake calculations were based on foods and beverages entered from the baseline three-day records. Nutrient contributions from dietary supplements were not separately quantified and therefore should not be interpreted as included in the reported intakes.

The analysis included total energy intake and intake of carbohydrates, protein, fat, saturated fatty acids (SFAs), monounsaturated fatty acids (MUFAs), polyunsaturated fatty acids (PUFAs), and dietary fibre. Micronutrients included vitamin A, folate, vitamin B_12_, vitamin C, vitamin D, calcium, magnesium, iron, and potassium.

Individual daily energy requirements were estimated by multiplying the basal metabolic rate (BMR) estimate provided by the InBody 770 by a physical activity level (PAL) selected according to weekly training volume and overall physical activity. A PAL of 1.8 was applied to athletes training approximately 4–7 h/week, whereas a PAL of 2.2 was applied to athletes training approximately 20 h/week [16]. Mean reported energy intake derived from the three-day dietary records was subsequently expressed as a percentage of the individually estimated energy requirement. As these calculations were based on device-derived BMR estimates and approximate PAL factors rather than direct measurements of total energy expenditure, the resulting values were used as practical comparative estimates and should not be interpreted as direct measures of energy availability.

Prespecified reference values were used to evaluate dietary intake. These values were primarily derived from the Czech rhythmic gymnastics coaching textbook published by Masaryk University in collaboration with the Czech Rhythmic Gymnastics Association [17] and were considered alongside the European Food Safety Authority (EFSA) dietary reference values [18]. This source was selected because it provides discipline-specific nutritional guidance within the Czech rhythmic gymnastics’ context. The reference values were therefore used as standardised sport-oriented benchmarks for descriptive comparisons rather than as individual age-specific nutritional requirements (Table 1).

The same prespecified sport-oriented reference values were applied to both junior and senior athletes to maintain consistency with the original study protocol and to allow standardised descriptive comparisons across competitive age categories. These benchmarks were not age-weighted and should not be interpreted as individual age-specific dietary reference intakes. For example, the EFSA population reference intake for calcium is 1150 mg/day for adolescents aged 11–17 years and 1000 mg/day for young adults aged 18–24 years [18]. Since the senior competitive category in the present study included athletes aged 16–20 years, age-related differences may exist within this group. Therefore, the original sport-oriented benchmarks should be interpreted as common study-specific comparison targets rather than individual age-adjusted nutrient requirements.

### 2.4. Individualised Nutrition Intervention

The nutrition intervention comprised two components. First, at the baseline, each athlete received an educational leaflet on energy and macronutrient intake, hydration, vitamins and minerals, and the potential consequences of low energy availability and relative energy deficiency in sport (RED-S). The leaflet covered principles consistent with the sports-nutrition position statement and IOC RED-S guidance already cited in the manuscript [3,7], together with supporting material on recovery nutrition and nutrient reference values [16,17]. This was an educational resource, not a standardised or validated behaviour-change intervention.

Second, following analysis of the baseline three-day food record and body-composition assessment, each athlete received individualised written nutrition recommendations by a dietitian and certified sports nutritionist. Recommendations were tailored to the dietary and body-composition findings of each athlete and focused on supporting energy intake where the reported intake was low relative to the estimated requirements, addressing intakes below the selected nutritional benchmarks, and modifying potentially inappropriate eating habits.

Athletes were advised to follow the individualised recommendations for approximately three months. The original follow-up protocol specified a second body-composition assessment only. A repeated food record, objective adherence measure, and follow-up nutrition-knowledge assessment were not included. Accordingly, the present study can describe body-composition differences between the two visits but cannot establish whether dietary intake changed, whether the recommendations were followed, or whether any observed change was caused by counselling.

### 2.5. Body-Composition Assessment

Body composition was assessed using an InBody 770 multifrequency, eight-electrode bioelectrical impedance analyser (InBody Co., Ltd., Seoul, Republic of Korea). Body height was measured using a SECA 213 stadiometer (SECA, Hamburg, Germany).

Each participant underwent two measurements. Baseline measurements (M1) were conducted in November or early December, and follow-up measurements (M2) were performed in February, approximately three months after the baseline assessment and nutritional intervention.

The analysed body-composition variables were body weight, skeletal muscle mass, fat mass, body fat percentage, body mass index (BMI), fat-free mass, total body water, and the extracellular-to-total body water ratio (ECW/TBW).

To improve the standardisation and reproducibility of bioimpedance measurements, participants received pre-measurement instructions. Measurements were performed barefoot and in underwear, with the participants standing in the standardised measurement position required by the device. Athletes were instructed to attend the measurement at least 2 h after their last meal and to avoid strenuous physical activity before measurement, to avoid caffeine on the day of measurement, to empty their bladder before testing, and to arrive adequately hydrated. Where possible, athletes with a menstrual cycle were advised to undergo measurement outside menstruation, preferentially during the first half of the cycle. Participants were required to be free from acute illness or injury that could materially affect the measurement.

The same type of device and standardised measurement conditions were used for both the baseline and follow-up assessments.

### 2.6. Study Component 2: Nationwide Questionnaire Survey

To complement the small intervention cohort, a study-specific 44-item online questionnaire was developed in Microsoft Forms to explore nutrition-related behaviours and experiences among competitive rhythmic gymnasts in the Czech Republic. The questionnaire covered six principal domains: anthropometric and sport-related characteristics; dietary habits; sports nutrition knowledge and supplement use; attitudes towards eating and body mass; recovery and body-composition practices; and interest in professional nutrition support. No formal content-validity assessment, psychometric validation, or reliability testing was conducted. Accordingly, the questionnaire was intended for descriptive purposes rather than clinical diagnosis or validated prevalence estimation.

Participants were recruited through voluntary online dissemination using personal contacts, email communication with rhythmic-gymnastics clubs, and relevant Facebook groups. Recruitment was non-probability based. As the number of eligible athletes receiving the invitation was not documented, a response rate could not be calculated. The final sample comprised 50 respondents (18 juniors and 32 seniors) and should not be considered representative of all Czech competitive rhythmic gymnasts.

More specifically, the questionnaire collected information on age, height, body mass, years of participation in rhythmic gymnastics, competitive category, weekly training frequency and duration, participation in additional sports, number and regularity of meals, breakfast and snack consumption, hunger and food intake after training, monitoring of energy or nutrient intake, habitual fluid intake and beverage choices, consumption of sweets, perceived sports-nutrition knowledge, sources influencing dietary behaviour, dietary-supplement use, awareness of RED-S, perceived pressure to maintain or reduce body mass, intentional food restriction for performance or appearance, self-reported history of eating-related problems, frequency of body-composition assessment, perceived influence of nutrition on athletic performance, previous sports injuries, recovery practices, and interest in access to a nutrition professional within the sports club. Body weight and height were self-reported by the questionnaire respondents and were not independently verified through anthropometric measurements.

Most items were closed-ended and used predefined response categories, including yes/no responses, frequency categories, multiple-choice questions, and ordinal response scales. Eight questions were open-ended and enabled respondents to provide additional information regarding dietary behaviour, post-training intake, body-related experiences, supplements, previous injuries, and nutrition-related interests.

An information and consent statement was presented at the beginning of the online questionnaire. Participation was voluntary, and informed consent was obtained from all adult respondents. For participants younger than 18 years, informed consent was obtained from a parent or legal guardian prior to participation. Questionnaire data were analysed descriptively. Absolute frequencies (*n*) and relative frequencies (%) were calculated for individual response categories.

### 2.7. Statistical Analysis

Statistical analyses were performed using jamovi version 2.6 (The jamovi project, Sydney, Australia). Microsoft Excel 2021 (Microsoft Corporation, Redmond, WA, USA) was used for the preparation of tables and graphical presentation of the questionnaire results. Additional analyses of the proportions of athletes meeting the prespecified nutritional benchmarks, including Fisher’s exact tests and the Benjamini–Hochberg correction, were performed using Python version 3.13.5 (Python Software Foundation, Wilmington, DE, USA) with SciPy version 1.17.0.

Continuous variables were summarised using descriptive statistics, including mean, median, standard deviation (SD), minimum, and maximum values, as appropriate. Normality was assessed using the Shapiro–Wilk test, and homogeneity of variances between junior and senior athletes was evaluated using Levene’s test. Parametric tests were applied when their assumptions were satisfied; otherwise, appropriate non-parametric alternatives were used.

For each participant, mean daily nutrient intake was calculated from the three-day dietary record. These individual mean values were subsequently compared with the prespecified nutritional benchmarks separately for junior and senior athletes using one-sample *t*-tests or Wilcoxon signed-rank tests, depending on the distribution of the data. Thus, statistical comparisons were based on participant-level observations rather than a single aggregated group mean. These analyses assessed differences from the predefined study benchmarks and were not intended to establish individual age-specific dietary adequacy or biochemical nutrient deficiency.

Between-group differences in dietary intake, participant characteristics, and body-composition parameters were assessed using independent-samples *t*-tests or Mann–Whitney U tests, as appropriate. Welch’s *t*-test was applied when the assumption of homogeneity of variances was violated.

Changes in body composition between the baseline (M1) and follow-up (M2) were evaluated within each age category using paired-samples *t*-tests or Wilcoxon signed-rank tests when the normality assumption for paired differences was not satisfied. Individual change scores (Δ = M2 − M1) were also calculated and compared between junior and senior athletes using independent-samples *t*-tests, Welch’s *t*-tests, or Mann–Whitney U tests, depending on the distribution of the data and homogeneity of variances. For the exploratory between-group comparison of ECW/TBW change scores, a 95% confidence interval for the mean difference was additionally calculated using a pooled-variance independent-samples *t*-test.

Effect sizes were reported alongside *p*-values. Cohen’s d was used for parametric comparisons, with values of approximately 0.2, 0.5, and ≥0.8 interpreted as small, moderate, and large effects, respectively. Rank-biserial correlation (r) was used for non-parametric comparisons, with values of approximately 0.1, 0.3, and ≥0.5 interpreted as small, moderate, and large effects, respectively.

Additionally, the proportion of participants reaching each prespecified nutritional benchmark was calculated separately for junior and senior athletes using individual mean dietary intakes derived from the three-day food records. Between-group differences in these proportions were assessed using two-sided Fisher’s exact tests. To account for multiple comparisons across the 16 dietary components, the Benjamini–Hochberg procedure was applied to control the false discovery rate.

Questionnaire data were analysed descriptively using absolute and relative frequencies. No inferential statistical comparisons were performed for the questionnaire component.

All inferential analyses were conducted using a significance level of α = 0.05. No adjustment for multiple comparisons was applied to the original continuous-variable analyses, whereas the additional Fisher’s exact tests were adjusted using the Benjamini–Hochberg procedure. Accordingly, the original *p*-values were considered exploratory, and the results of the additional categorical comparisons were interpreted using the adjusted q-values. Given the pilot nature of the study and the small sample size, all findings were interpreted cautiously.

## 3. Results

### 3.1. Participant Characteristics

The intervention cohort comprised 14 female competitive rhythmic gymnasts (six juniors and eight seniors). Participant characteristics are summarised in Table 2. Seniors were older than juniors (17.4 ± 1.4 vs. 14.3 ± 0.5 years; Mann–Whitney U, *p* = 0.002), as expected from the predefined age categories. Height and body mass did not differ significantly between groups. Weekly training volume was numerically higher in seniors (18.0 ± 5.7 h/week) than in juniors (10.3 ± 7.6 h/week) but did not reach statistical significance (*p* = 0.051). Training volume among juniors showed substantial variability, ranging from 4 to 20 h/week.

### 3.2. Dietary Intake Relative to Prespecified Benchmarks

#### 3.2.1. Energy and Macronutrient Intake

Mean energy intake was 63.7 ± 18.2% of the individually estimated requirement in juniors and 67.1 ± 16.5% in seniors, with no between-group difference (*p* = 0.718). In both groups, energy intake was significantly below the estimated requirement (*p* = 0.004 and *p* < 0.001, respectively). Carbohydrate intake was also below the prespecified reference of 5.5 g/kg body mass/day in juniors (3.91 ± 0.98 g/kg/day, *p* = 0.011) and seniors (4.10 ± 1.24 g/kg/day, *p* = 0.015). Dietary fibre intake was markedly below 25 g/day in both groups (10.10 ± 4.25 and 13.30 ± 5.59 g/day; both *p* < 0.001).

Protein intake did not differ from the 1.25 g/kg/day reference in juniors (1.27 ± 0.43 g/kg/day, *p* = 0.911), whereas seniors consumed significantly more protein than the reference value (1.68 ± 0.30 g/kg/day, *p* = 0.004). Total fat intake was 34.0 ± 6.06% of energy in juniors and 35.8 ± 3.98% in seniors; only the senior group differed significantly from the 30% reference (*p* = 0.004). Between groups, juniors had a higher proportion of saturated fatty acids (8.58 ± 3.01% vs. 6.07 ± 0.62% of energy, *p* = 0.039), whereas seniors had a higher proportion of monounsaturated fatty acids (4.01 ± 1.32% vs. 2.46 ± 2.63% of energy, Mann–Whitney U, *p* = 0.045). No other between-group differences in energy or macronutrient intake reached statistical significance (Table 3).

#### 3.2.2. Micronutrient Intake

Micronutrient intake varied considerably among participants, particularly for vitamin A and folate. No statistically significant differences in micronutrient intake were observed between junior and senior athletes (Table 4).

Compared with the prespecified study benchmarks, the lowest reported intakes relative to the reference values were observed for vitamin D, calcium, magnesium, and potassium in both age categories. Iron intake was significantly below the study benchmark in juniors but not in seniors. In contrast, reported intakes of vitamin A, vitamin B_12_, vitamin C, and folate did not differ significantly from their respective benchmarks in either group. These results describe dietary intake relative to prespecified reference values and do not establish individual biochemical nutrient deficiencies.

**Figure 1:**
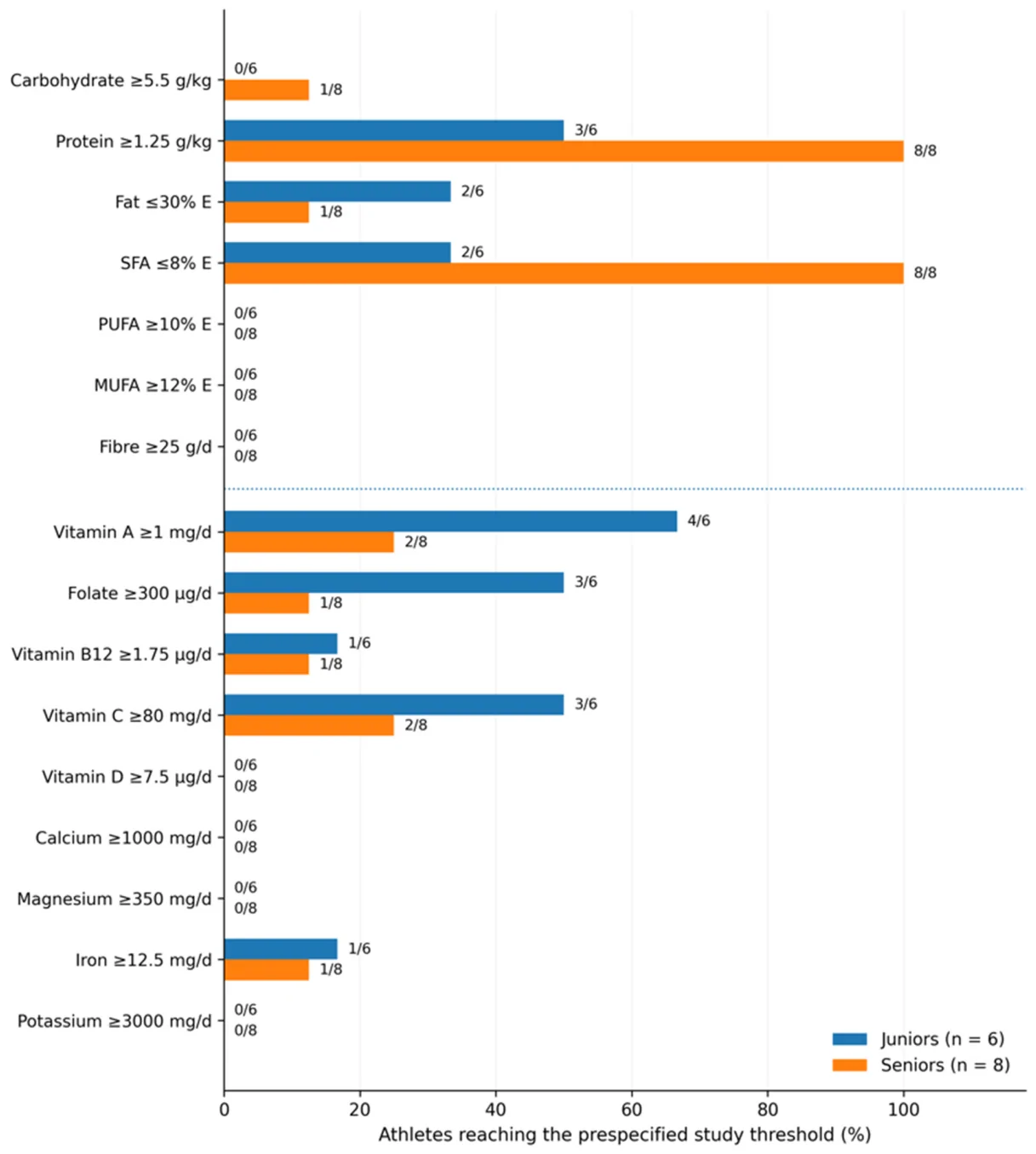
Proportion of junior and senior rhythmic gymnasts reaching the prespecified nutritional benchmarks. Note: Bars represent percentages of athletes reaching the respective study-specific thresholds, with absolute numbers shown as *n*/N (juniors, *n* = 6; seniors, *n* = 8). For total fat and saturated fatty acids, intakes of ≤30% and ≤8% of total energy, respectively, were classified as reaching the thresholds. For the remaining dietary components, intakes at or above the prespecified values were counted. Total fat, PUFA, and MUFA thresholds represent operational study targets rather than established adequacy ranges. Energy adequacy was not included because individual estimated energy requirements were unavailable in the dataset used for these additional analyses. The percentages do not represent compliance with individual age-specific EFSA reference values. SFA, saturated fatty acid; PUFA, polyunsaturated fatty acid; MUFA, monounsaturated fatty acid.

Figure 1 presents the proportion of junior and senior gymnasts reaching the prespecified nutritional benchmarks based on individual mean dietary intakes. None of the participants reached the study benchmarks for dietary fibre, vitamin D, calcium, magnesium, or potassium. The carbohydrate benchmark was reached by none of the juniors and only one senior athlete (12.5%), whereas the protein benchmark was reached by 50.0% of juniors and 100% of seniors.

Exploratory between-group comparisons using Fisher’s exact test identified a nominally significant difference only in the proportion of athletes with saturated fatty acid intake at or below the prespecified threshold of 8% of total energy intake (33.3% in juniors vs. 100% in seniors; unadjusted *p* = 0.015). However, this difference did not remain statistically significant after adjustment for multiple comparisons using the Benjamini–Hochberg procedure (adjusted q = 0.240). No other between-group comparisons were statistically significant. Given the small sample size, these findings should be interpreted cautiously. The reported proportions represent attainment of the prespecified study benchmarks rather than compliance with individual age-specific dietary reference values.

Overall, the reported energy and carbohydrate intakes in both age categories were below the prespecified comparative targets, while fibre, vitamin D, calcium, magnesium, and potassium intakes were also low relative to the selected protocol benchmarks. These deviations from study-specific targets should not be interpreted as confirmed individual nutritional inadequacies, particularly given the age range and short dietary recording period.

### 3.3. Body Composition at Baseline and Follow-Up

Body-composition values at the baseline (M1) and after approximately three months of individualised nutrition recommendations (M2) are shown in Table 5. No statistically significant within-group changes were observed for body mass, skeletal muscle mass, fat mass, body fat percentage, BMI, total body water, or fat-free mass in either age category. In juniors, body mass remained essentially unchanged (51.7 ± 2.8 to 51.6 ± 3.5 kg), as did skeletal muscle mass (22.9 ± 1.2 to 22.9 ± 1.4 kg), body fat percentage (18.3 ± 1.6% to 18.2 ± 1.4%), and BMI (19.3 ± 1.3 to 19.1 ± 1.4 kg/m^2^). ECW/TBW decreased from 0.383 ± 0.007 to 0.380 ± 0.004; however, the within-group change was not statistically significant (*p* = 0.055; d = 1.015).

In seniors, body mass remained stable (54.6 ± 4.1 vs. 54.8 ± 3.9 kg). Skeletal muscle mass increased numerically from 25.3 ± 2.3 to 26.4 ± 4.7 kg, while body fat percentage increased from 15.7 ± 2.2% to 16.5 ± 2.1%; neither change was statistically significant. Fat mass and body fat percentage both showed moderate within-group effect sizes (d = −0.628; *p* = 0.119), but the small sample size precluded firm conclusions.

Comparing individual change scores (Δ = M2 − M1) between age categories revealed a nominally significant difference only for ECW/TBW (Table 6). Juniors showed a mean decrease of −0.00350, whereas seniors showed a mean increase of 0.00075. The mean between-group difference (juniors minus seniors) was −0.00425 (95% CI: −0.00753 to −0.00097; unadjusted *p* = 0.015; Cohen’s d = −1.527). This finding was exploratory and should be interpreted cautiously given the small sample size and multiple unadjusted comparisons. No statistically significant between-group differences were observed for the remaining body-composition change scores.

### 3.4. Nationwide Questionnaire Survey

#### 3.4.1. Participant Characteristics

The nationwide questionnaire was completed by 50 rhythmic gymnasts (18 juniors and 32 seniors). Juniors had a mean age of 14.3 years, with self-reported body mass and height averaging 47.2 kg and 161.6 cm, respectively. Corresponding values in seniors were 18.4 years, 55.6 kg, and 167.4 cm. Participation in rhythmic gymnastics averaged 8 years in juniors and 13 years in seniors, and the mean training session duration was about 4 h in both groups. Most respondents trained 4–6 times per week (72%), while 28% trained 2–3 times per week; respondents also reported at least one two-a-day training sessions per week. Running was the most frequently reported additional activity (26%), followed by gym-based exercise (20%), swimming (14%), and in-line skating (11%); 11% reported no additional sport and 17% reported another activity.

#### 3.4.2. Dietary Habits, Hydration, Supplements, and Nutrition Knowledge

Most respondents reported consuming four or five meals per day (32% and 36%), while 22% consumed three meals, 6% six meals, and 4% only two meals. Eighty-eight percent described their eating pattern as regular or mostly regular. Breakfast was consumed daily by 64% of respondents; 20% consumed breakfast occasionally, 10% rarely, and 6% never. Snacks between main meals were consumed regularly by 40% and occasionally by 44%; among the snack consumers, two snacks per day was the most common frequency (59%). Fruit, vegetables, and yoghurt were the most frequently reported snack choices.

A notable mismatch was observed between post-exercise hunger and recovery eating. Although 68% of respondents reported substantial hunger after training, only 22% reported regularly consuming a post-training snack; 32% did so occasionally, 28% rarely, and 18% never. In contrast, 94% reported eating dinner every day. Daily fluid intake was most commonly 1.5–2 L (74%); 20% reported 2–3 L and 6% less than 1 L. All respondents reported drinking water, while tea (*n* = 23), fruit beverages (*n* = 14), isotonic drinks (*n* = 10), and coffee (*n* = 10) were also reported; no respondent reported alcohol consumption.

Dietary supplements were used by 42% of respondents, whereas 58% reported no supplement use. Among users, vitamins (*n* = 12) and minerals, particularly magnesium, zinc, calcium, and selenium (*n* = 11), were the most frequently reported categories, followed by omega-3 fatty acids (*n* = 3) and other products such as collagen, chlorella, curcumin, or electrolytes (*n* = 5). Only 12% of respondents reported regularly monitoring energy or nutrient intake; 32% did so occasionally, 42% rarely, and 14% never. Sweet cravings were reported by 76%; sweets were consumed regularly by 40%, occasionally by 50%, and rarely by 10%.

Self-rated sports-nutrition knowledge was limited: 64% reported only partial knowledge, 22% considered their knowledge sufficient, and 14% reported almost no knowledge. Parents (*n* = 28) and coaches (*n* = 23) were the most frequently reported influences on eating behaviour, followed by social media (*n* = 17). Only four respondents reported influence from a nutrition professional.

#### 3.4.3. RED-S Awareness, Weight-Control Behaviours, and Eating-Related Concerns

Awareness of RED-S was low: 56% of respondents had never heard of the syndrome, 32% had heard the term but did not know its meaning, and only 12% reported knowing what RED-S was. A total of 68% reported having experienced pressure to maintain or reduce body mass because of rhythmic gymnastics. In addition, 60% reported intentionally restricting food intake for performance or appearance-related reasons. Open-ended responses indicated that restriction most commonly involved reducing the total food quantity or portion size, skipping meals or snacks, and limiting sweets, bread, or carbohydrate-rich side dishes; several respondents also described dieting or deliberate attempts to lose body mass.

Although most open-ended responses described a positive or neutral relationship with food and body image, a smaller proportion reported guilt after eating, dissatisfaction with their body, or periods of a more problematic relationship with food. A self-reported history of an eating disorder was reported by 10% of respondents (*n* = 5), whereas 90% reported no such history. Among those reporting an eating-disorder history, anorexia nervosa, bulimia nervosa, and binge-eating episodes were all represented.

#### 3.4.4. Body-Composition Monitoring, Performance, Injury, Recovery, and Professional Support

Body-composition monitoring was relatively uncommon. Approximately one-third of respondents (34%) reported undergoing body-composition assessment once per year, 28% only occasionally, and 30% had never undergone such assessment; only 8% reported being assessed several times per year.

Despite the limited use of body-composition monitoring, most respondents recognised the importance of nutrition for sport performance, with 78% reporting that nutrition influenced their athletic performance. A history of sports injury was reported by 70% of respondents. Open-ended responses most frequently described lower-limb injuries, including ankle sprains and ligament injuries, muscle tears, knee injuries, and fractures, including stress fractures; spinal injuries were reported less frequently. Physiotherapy and rehabilitation were the most commonly reported treatment approaches, often accompanied by rest or a temporary reduction in training.

Recovery practices were dominated by sleep, followed by massage, whereas sauna and other strategies were reported less frequently. Nutritional recovery strategies appeared to be less consistently implemented, as reflected by the previously described discrepancy between post-training hunger and regular post-training snack consumption.

Importantly, 72% of respondents indicated that they would welcome access to a nutrition professional within their sports club. Together with the finding that 78% perceived nutrition as relevant to performance, this suggests a high level of interest in nutrition-related support among the surveyed gymnasts.

**Figure 2:**
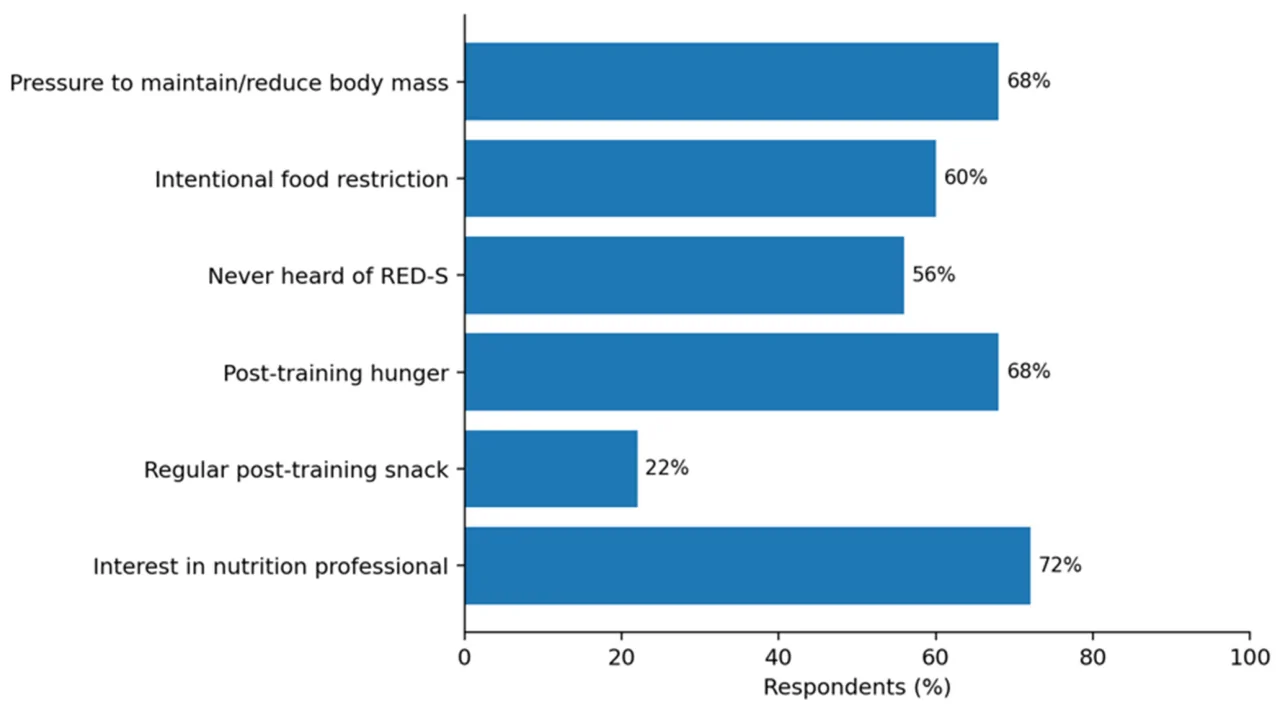
Selected nutrition-related behaviours, RED-S awareness, and interest in professional nutrition support among Czech competitive rhythmic gymnasts. The presented proportions refer to the overall questionnaire sample (*n* = 50), comprising 18 juniors and 32 seniors. RED-S, relative energy deficiency in sport.

A selected overview of the most relevant behavioural findings is presented in Figure 2.

## 4. Discussion

This exploratory pilot study found low reported energy and carbohydrate intakes and several low micronutrient intakes relative to fixed protocol benchmarks, alongside frequent self-reported weight-control behaviours in a separate questionnaire sample. Two body-composition measurements approximately three months apart did not show statistically significant within-group differences. Importantly, the counselling period was not accompanied by reassessment of dietary intake or intervention adherence. The data therefore support descriptive identification of potential nutrition-support needs, not causal conclusions about the effectiveness of counselling or diagnoses of nutrient deficiency, LEA, or RED-S. Importantly, the dietary and body-composition assessments and the nationwide questionnaire represented separate study components with independently analysed samples. Therefore, associations between individual dietary intakes, body-composition outcomes, and questionnaire-reported behaviours cannot be established from the present data.

The low reported energy intake observed in both age groups is consistent with previous findings in rhythmic gymnastics. Silva and Paiva (2014) reported inadequate energy intake and low energy availability among female gymnasts before an international competition [9], while Miteva et al. (2019) similarly described insufficient energy intake and restrictive dietary practices among elite Bulgarian rhythmic gymnasts [10]. Villa et al. (2021) also identified a risk of low energy availability in elite-level competitive rhythmic gymnasts [11]. These findings are particularly relevant given the substantial training volume in the present cohort, which averaged 10.3 h/week in juniors and 18.0 h/week in seniors. Persistent mismatch between energy intake and exercise demands may increase the likelihood of low energy availability (LEA), which represents the underlying mechanism of RED-S and may adversely affect reproductive and endocrine function, bone health, immunity, gastrointestinal and cardiovascular function, psychological health, recovery, and athletic performance [7]. However, an important distinction must be made between low reported energy intake and clinically established LEA. Exercise energy expenditure was not directly measured in the present study, and energy availability could therefore not be calculated. Consequently, the current findings should be interpreted as evidence of low reported energy intake relative to estimated requirements and potential under-fuelling, rather than as a diagnosis of LEA or RED-S.

Carbohydrate intake was another parameter falling below the prespecified study benchmark. Mean intake was 3.91 g/kg/day in juniors and 4.10 g/kg/day in seniors, significantly below the prespecified reference value of 5.5 g/kg/day in both groups. Similar patterns have previously been reported among rhythmic gymnasts [9,10]. This finding is also consistent with the behavioural data from the present questionnaire, in which 60% of respondents reported having intentionally restricted food intake for performance- or appearance-related reasons. Open-ended responses indicated that restriction commonly involved reducing overall food quantity, skipping meals or snacks, and limiting bread, sweets, or carbohydrate-rich side dishes. This is of particular concern because carbohydrate availability contributes to the maintenance of training intensity and the restoration of muscle glycogen between repeated exercise sessions [3]. In athletes performing frequent and prolonged training, as observed particularly among the senior gymnasts, chronically insufficient carbohydrate availability may therefore compromise recovery and the ability to sustain training quality. From a practical sports-nutrition perspective, improving total energy and carbohydrate availability may consequently be more important than pursuing further reductions in body mass or body fat.

The proportional contribution of fat to total energy intake was 34.0% in juniors and 35.8% in seniors, with the senior group significantly exceeding the prespecified 30% reference value. Similar proportional fat intakes have been described in rhythmic gymnasts [9,10]. However, this result should not automatically be interpreted as excessive absolute fat consumption. In the context of low reported total energy and carbohydrate intake, a higher proportional contribution from fat may partly reflect a reduced contribution from carbohydrates. Differences in fatty-acid composition between the two age categories should likewise be interpreted cautiously because dietary intake was assessed only over three days and showed considerable inter-individual variability.

Protein intake was comparatively more favourable. Juniors consumed 1.27 g/kg/day and seniors 1.68 g/kg/day. According to the prespecified study reference of 1.25 g/kg/day [17], mean protein intake met the prespecified benchmark, with senior athletes consuming significantly more than this threshold. Nevertheless, broader sports-nutrition recommendations commonly suggest approximately 1.4–2.0 g/kg/day for exercising individuals, depending on training characteristics, energy availability, and training goals [19]. From this perspective, the senior group was within the recommended range, whereas mean intake among juniors was somewhat lower. This may be particularly relevant during adolescence, when dietary protein must support both exercise adaptation and normal growth and maturation. Importantly, adequate protein intake cannot compensate for insufficient total energy or carbohydrate intake; therefore, the overall dietary pattern should remain the primary focus rather than the intake of an isolated macronutrient.

Dietary fibre intake was comparatively low in both age groups (10.1 g/day in juniors and 13.3 g/day in seniors) in relation to the original 25 g/day benchmark. However, this benchmark does not capture all age-specific considerations, and the short dietary recording period limits conclusions about habitual adequacy. Villa et al. (2021) reported relatively low fibre intake in elite rhythmic gymnasts [11]. Current sports-nutrition literature also emphasises that fibre needs to be balanced with gastrointestinal comfort around intensive training [20]. Overall dietary quality and the inclusion of nutrient-dense carbohydrate foods are appropriate practical considerations, while this dataset cannot identify which individuals failed age-specific recommendations.

Among the micronutrients assessed, vitamin D showed one of the largest discrepancies from the prespecified reference value. Mean dietary intake was only 0.72 μg/day in juniors and 1.83 μg/day in seniors. Low dietary vitamin D intake has also been reported among elite gymnasts [9]. This finding is particularly relevant in an indoor sport, because vitamin D status is influenced not only by dietary intake, but also by cutaneous synthesis and seasonal ultraviolet exposure [21]. A large recent study in the Czech Republic reported a high prevalence of low vitamin D status in the general population [22], while a systematic review and meta-analysis identified vitamin D insufficiency as a common finding among elite athletes, including adolescents, with seasonal variation representing an important risk factor [23]. Measurements in the present study were performed during November or early December and again in February, corresponding to a period of limited ultraviolet exposure in Central Europe. Nevertheless, serum 25-hydroxyvitamin D was not measured, and the present findings therefore demonstrate very low dietary vitamin D intake, not biochemical vitamin D deficiency. This distinction is important when considering supplementation. The results support dietary assessment, and where clinically appropriate, a biochemical evaluation of vitamin D status rather than routine high-dose supplementation based solely on food-record data.

Reported calcium intake was substantially below the prespecified study benchmark, averaging 396 mg/day in juniors and 423 mg/day in seniors, corresponding to approximately 40% of the original 1000 mg/day reference value. Similar inadequacies have previously been reported among rhythmic gymnasts [9,11] and adolescent gymnasts [12]. This finding is especially important in younger athletes because adolescence represents a critical period for bone-mass accrual. Adequate calcium intake must also be considered in the context of energy availability and vitamin D status, all of which contribute to bone health. The simultaneous observation of low reported energy intake and very low dietary calcium and vitamin D intake therefore warrants attention in a sport characterised by repetitive mechanical loading and a recognised risk of bone stress injury [7]. However, bone mineral density and biochemical vitamin D status were not assessed in the present study, and no direct conclusions regarding bone health can be drawn from the dietary data alone.

Magnesium intake was similarly low, averaging 162 mg/day in juniors and 165 mg/day in seniors compared with the 350 mg/day reference value. A systematic review and meta-analysis by Zhang et al. (2023) reported lower serum magnesium concentrations and greater 24-h urinary magnesium excretion among athletes despite higher dietary magnesium intake than in non-athletes, suggesting that physical training may influence magnesium balance [24]. This does not, however, mean that the present participants had biochemical magnesium deficiency, because no laboratory assessment was performed. The finding should instead be interpreted as reported magnesium intake below the prespecified study benchmark.

Potassium intake was also substantially below the prespecified reference value, reaching 1433 mg/day in juniors and 1619 mg/day in seniors. Villa et al. (2021) previously reported inadequate potassium intake among elite rhythmic gymnasts [11], while Aguiló et al. (2021) observed persistent nutritional inadequacies during a nutrition-education programme in young artistic gymnasts [25]. Together with the low fibre intake, the present potassium findings may reflect insufficient overall intake of nutrient-dense carbohydrate-containing foods such as fruits, vegetables, legumes, potatoes, and whole-grain products. Accordingly, an intervention emphasising overall food quality and sufficient energy availability may be more appropriate than isolated micronutrient supplementation.

Iron intake deserves consideration. Mean intake was 7.55 mg/day in juniors and 9.07 mg/day in seniors. Importantly, intake was significantly below the prespecified 12.5 mg/day reference value in juniors, whereas the difference from the reference value was not statistically significant among seniors; no significant between-group difference was observed. Female athletes may be particularly susceptible to iron depletion as a result of menstrual blood loss, exercise-associated iron losses, growth requirements, and insufficient dietary intake [26,27]. Nevertheless, dietary iron intake cannot establish iron status. Ferritin, haemoglobin, transferrin saturation, inflammatory markers, and other biochemical indicators were not assessed in the present study. Previous research indicates that dietary strategies may contribute to improvements in iron status in female athletes [28], supporting biochemical evaluation where low intake or clinical risk factors are identified rather than assuming deficiency from dietary records alone.

The body-composition results provided an important complement to the dietary findings. Baseline body-fat percentage was 18.3% in juniors and 15.7% in seniors, and BMI averaged 19.3 and 19.7 kg/m^2^, respectively. Comparable relatively low body-fat values have been reported in elite rhythmic gymnasts [10,11]. However, the interpretation of body composition in aesthetic sports requires caution. Current best-practice recommendations emphasise that there is no universally optimal body-fat percentage applicable to all athletes and that body-composition targets should be individualised according to health, developmental stage, nutritional status, and sporting requirements [8]. This is particularly important in adolescents, for whom growth and maturation should take priority over attempts to achieve an arbitrarily lean physique. Furthermore, BMI alone provides limited information in athletes because it does not distinguish fat mass from fat-free mass.

No statistically significant within-group changes were observed in body mass, skeletal muscle mass, fat mass, body fat percentage, BMI, total body water, fat-free mass, or ECW/TBW following approximately three months of individualised nutrition recommendations. The observed stability of body composition does not permit evaluation of the effectiveness of nutritional counselling. The intervention was relatively short, no non-intervention control group was included, adherence to the recommendations was not objectively monitored, and dietary intake was not reassessed after the intervention. Consequently, it is not possible to determine whether the athletes modified their actual dietary intake, nutrition knowledge, or recovery practices despite the stability of body composition. Nutrition-education interventions in athletes show heterogeneous outcomes, with some evidence of improvements in nutritional knowledge and dietary behaviours but less consistent effects on physiological outcomes [14]. In young athletes, remotely delivered sports-nutrition education has also been shown to influence nutrition knowledge and aspects of dietary intake [29], while a longer education programme among young artistic gymnasts produced improvements in some dietary variables without substantial changes in most anthropometric outcomes [25]. Together, these findings illustrate the limitations of using body composition alone to assess short-term outcomes following nutrition education.

The only nominally significant difference in change scores between age categories was observed for ECW/TBW. Juniors showed a mean decrease of −0.00350, whereas seniors showed a small mean increase of 0.00075 (unadjusted *p* = 0.015). However, the within-group change among juniors was not statistically significant (*p* = 0.055). Because bioelectrical-impedance-derived fluid indices are influenced by hydration and other pre-measurement conditions, and because the intervention sample was small, this isolated between-group finding should be interpreted with caution. The present data do not support attributing the ECW/TBW change specifically to improved hydration or to the nutrition intervention. Accordingly, the original hypothesis that junior athletes would demonstrate a greater overall body-composition response to the intervention was not supported.

The nationwide questionnaire provides important behavioural context for these nutritional findings. One of the most actionable observations was the mismatch between post-exercise hunger and recovery eating. Although 68% of respondents reported substantial hunger following training, only 22% regularly consumed a post-training snack, while 46% reported consuming one rarely or never. Appropriate post-exercise nutrition is relevant for the restoration of glycogen and the provision of amino acids for muscle repair and adaptation, especially when recovery time between training sessions is limited [4]. Previous research also suggests that knowledge and adherence to post-exercise nutrition recommendations may remain suboptimal even among regularly training athletes [30]. In the present sample, improving access to convenient post-training food therefore represents a practical intervention target. Rather than simply advising athletes to “eat more”, individualised recommendations could focus on realistic combinations of carbohydrate and high-quality protein adapted to training duration, the timing of the subsequent meal, gastrointestinal tolerance, and individual energy requirements.

Self-reported fluid intake should be interpreted more cautiously. Most respondents reported consuming approximately 1.5–2 L/day, while 20% reported 2–3 L/day and 6% less than 1 L/day. Although these values may appear relatively low in the context of prolonged training, daily fluid volume alone cannot be used to diagnose inadequate hydration. Fluid requirements differ substantially according to sweat rate, environmental conditions, training duration, body size, and fluid intake during exercise [3]. The questionnaire therefore supports the need for individualised hydration strategies rather than a universal daily fluid target. Where practical, these strategies could incorporate pre- and post-training body-mass changes and individual sweat losses.

The weight-control findings deserve particular attention. Among the respondents, 68% reported having experienced pressure to maintain or reduce body mass because of rhythmic gymnastics, and 60% reported intentional restriction of food intake for performance- or appearance-related reasons. These results are consistent with the broader literature indicating that aesthetic sports may create an environment in which performance expectations, body image, and dietary restriction become closely connected [5,6]. Miteva et al. (2019) likewise described restrictive weight-management practices among elite rhythmic gymnasts [10]. In Greek adolescent gymnasts, Kontele et al. (2022) observed greater eating-disorder symptoms and weight-related pressures among competitive than recreational gymnasts, with sport- and coach-related pressures associated with disordered-eating symptoms [31]. An exploratory study in Italian rhythmic gymnasts similarly identified substantial eating-disorder risk and emphasised the importance of monitoring and preventive strategies [32].

In the present survey, 10% of respondents reported a previous eating disorder. This finding should not be interpreted as the prevalence of clinically diagnosed eating disorders, because the questionnaire was study-specific and was not designed as a validated diagnostic instrument. Self-report may also be influenced by recall and social-desirability bias. Nevertheless, the combination of frequent weight-related pressure, intentional dietary restriction, low reported energy intake, and limited access to qualified nutrition professionals is clinically relevant even in the absence of a diagnosed eating disorder. Preventive interventions should therefore focus not only on nutrient intake but also on creating a sports environment in which discussions of body mass and body composition are handled appropriately.

Awareness of RED-S was particularly limited. More than half of the respondents (56%) had never heard of the syndrome, while only 12% reported knowing what it meant. This finding is consistent with the knowledge gap reported among adolescent gymnasts [13] and with evidence that awareness of LEA and RED-S among coaches may also be limited [33]. Besor et al. (2024) reported frequent weight-loss recommendations and restrictive dieting among adolescent acrobatic gymnasts and highlighted limited involvement of qualified nutrition professionals [34]. These findings are particularly relevant to the present study because parents and coaches were the two most frequently reported influences on athletes’ dietary behaviour, whereas only four respondents reported influence from a nutrition professional. The IOC consensus emphasises that the prevention and management of RED-S require education not only of athletes, but also of coaches and members of the wider athlete-support team [7]. Nutrition education in rhythmic gymnastics should therefore extend beyond the individual athlete and involve coaches, and particularly for adolescent athletes, parents.

Approximately 70% of questionnaire respondents reported at least one sports injury during their career, including lower-limb injuries and fractures, some of which were described as stress fractures. The cross-sectional questionnaire cannot establish whether these injuries were related to energy or nutrient availability, and injury history should not be used as evidence that RED-S was present in these participants. Nevertheless, LEA and RED-S are associated with impaired bone health and increased susceptibility to bone stress injuries [7,35]. Against this background, the simultaneous observation of low reported energy intake and very low calcium and vitamin D intake is clinically relevant, particularly during adolescence. Future assessment should therefore incorporate menstrual history and objective measures of bone and biochemical health rather than attempting to infer bone-health status from dietary intake or injury history alone.

An encouraging finding was the athletes’ receptiveness to nutrition support. Seventy-eight percent of respondents perceived nutrition as influencing athletic performance, and 72% would welcome access to a nutrition professional within their sports club. Athletes generally report a preference for practical nutrition education delivered by appropriately qualified professionals and incorporating individualised feedback [15]. A recent intervention among young artistic gymnasts and ballet dancers also demonstrated that structured education could improve nutrition-related knowledge [36]. Together with the current finding that parents and coaches strongly influence eating behaviour, these results indicate an opportunity to establish systematic, professionally supervised nutrition programmes within rhythmic-gymnastics clubs. Such programmes should emphasise health and sustainable performance rather than body mass alone.

### 4.1. Strengths and Limitations

A principal strength of the present study is the combination of several complementary approaches: three-day dietary assessment, repeated multifrequency bioelectrical impedance analysis, individualised nutrition recommendations, and a broader nationwide questionnaire. This design enabled reported dietary intakes relative to the study benchmarks to be considered alongside body-composition changes and the behavioural and educational environment in which nutrition-related decisions were made. The inclusion of both junior and senior athletes additionally provided information across two relevant stages of athletic and physiological development.

Several limitations require emphasis. The intervention cohort was small (*n* = 14), volunteer-recruited and uncontrolled, and no a priori sample-size calculation was conducted. Consequently, statistical power was limited, group comparisons were exploratory, and non-significant results do not establish equivalence or absence of an effect. Without a control group, any changes between visits cannot be disentangled from normal growth and maturation, variation in training load, or other time-dependent factors. Multiple unadjusted comparisons additionally increase the possibility of chance findings, including the isolated ECW/TBW result.

The approximately three-month intervention period may also have been too short to detect meaningful physiological changes in body composition. Furthermore, baseline measurements were performed in November or early December and follow-up measurements in February, meaning that the intervention included the Christmas holiday period. Changes in training schedules, food availability, eating patterns, and habitual routines during this period may have influenced both adherence and physiological outcomes.

A three-day recording period may not adequately represent habitual dietary intake, particularly for micronutrients with substantial day-to-day variability. Consequently, the reported values should be regarded as short-term dietary estimates rather than definitive measures of long-term nutritional adequacy.

These limitations may be particularly relevant in athletes who experience pressure regarding body mass. Estimated energy requirements were derived from device-provided BMR estimates and PAL factors. Consequently, energy availability could not be calculated, and the present results cannot establish the presence of LEA or RED-S.

Dietary intake was measured only at the baseline. The follow-up protocol prioritised a second body-composition measurement; the diet record and objective adherence assessment were not repeated. Therefore, dietary change, compliance with recommendations, and effects of counselling on nutritional adequacy cannot be established. The study also did not quantify dietary supplement contributions to total nutrient exposure in the intervention cohort or assess nutrition knowledge longitudinally.

Body composition was assessed using multifrequency bioelectrical impedance analysis. Although this method is practical for field-based monitoring and standardised pre-measurement instructions were provided, bioimpedance measurements remain influenced by hydration status and other pre-measurement conditions. This is particularly relevant to interpretation of the ECW/TBW findings [8].

Biochemical outcomes (including iron status and serum 25-hydroxyvitamin D), menstrual function, hormonal markers, and bone mineral density were not assessed. Consequently, low reported dietary intake relative to a study benchmark cannot establish clinical nutrient deficiency, LEA, or RED-S. Furthermore, uniform sport-oriented benchmarks were applied to athletes aged 13–20 years without individual adjustment for age, growth, biological maturation, or training demands. Consequently, an intake below a selected benchmark indicates that a study-specific target was not reached but does not necessarily establish individual nutritional inadequacy. Although Figure 1 illustrates attainment of these thresholds, it cannot demonstrate compliance with individual age-specific dietary reference values.

Nutrient-density comparisons expressed per 1000 kcal were also not included in the original analytical framework. The study-specific nationwide questionnaire was self-reported, non-randomly recruited, and not formally validated; its findings should therefore be interpreted descriptively rather than diagnostically. These limitations, together with potential errors in dietary recording and food-composition data, constrain the generalisability and interpretation of the findings. Furthermore, body weight and height collected through the questionnaire were self-reported and could not be independently validated against objective anthropometric measurements. As only aggregated questionnaire results were available for additional analyses, potential reporting discrepancies and age-stratified differences in the selected questionnaire findings could not be assessed. The voluntary, non-probability recruitment process may have introduced selection bias, as athletes with greater interest in nutrition or weight-related concerns may have been more likely to participate.

### 4.2. Practical Implications

The present findings support a practical emphasis on adequate fuelling, carbohydrate intake, post-exercise recovery, and overall dietary quality rather than further reductions in body mass or body fat. Low reported intakes of several micronutrients should primarily prompt improvement in dietary quality, and where clinically indicated, targeted biochemical assessment rather than routine supplementation based solely on dietary records.

Nutrition education should extend beyond the individual athlete and involve coaches and parents, particularly in adolescent gymnasts. Qualified sports-nutrition professionals may play an important role in promoting appropriate fuelling, safe body-composition practices, RED-S awareness, and early recognition of restrictive or disordered eating behaviours. Body-composition assessment should be interpreted in the context of maturation, health, nutritional status, and psychological well-being rather than as an isolated performance target [8].

### 4.3. Future Directions

Future studies should confirm these pilot findings in larger, multicentre cohorts with longer follow-up, and where feasible, a comparison group. Repeated dietary assessment, objective measures of energy expenditure and adherence, and inclusion of training-load, performance, menstrual, biochemical, and bone-health outcomes would allow a more comprehensive evaluation of the effects of nutrition interventions. Future studies should also incorporate individual age-specific dietary reference values and nutrient-density analyses expressed per 1000 kcal to provide a more comprehensive comparison of dietary intake between competitive age categories.

Validated instruments for disordered eating, body-image concerns, and RED-S risk should also be incorporated. Longer-term interventions involving qualified sports dietitians, coaches, and parents may help determine whether improved nutritional adequacy translates into better recovery, athlete health, and sustainable performance.

## 5. Conclusions

This small pilot study described low reported dietary energy and carbohydrate intakes and several low micronutrient intakes relative to its original, non-age-specific protocol benchmarks in Czech competitive rhythmic gymnasts. A separate questionnaire identified substantial self-reported weight-related pressure, food restriction, and limited awareness of relative energy deficiency in sport. No statistically significant within-group body-composition changes were observed across two measurements approximately three months apart. Because no control group was included and dietary intake and adherence were not reassessed, these observations cannot establish the effectiveness of nutritional counselling. Importantly, intakes below the selected benchmarks do not necessarily indicate individual nutritional inadequacy, given age-related differences in growth, maturation, and nutrient requirements across athletes aged 13–20 years. Larger controlled studies incorporating repeated dietary assessment and age-appropriate reference values are warranted; meanwhile, qualified athlete-centred nutrition support appears appropriate.

## Figures and Tables

**Table 1:** Prespecified reference values used for comparisons of dietary intake with the study benchmarks [17].

Nutrient	Reference Value
Carbohydrate	5.5 g/kg body mass/day
Protein	1.25 g/kg body mass/day
Fat	30% of total energy intake
SFAs	8% of total energy intake
PUFAs	10% of total energy intake
MUFAs	12% of total energy intake
Dietary fibre	25 g/day
Vitamin A	1 mg/day
Folate	300 μg/day
Vitamin B_12_	1.75 μg/day
Vitamin C	80 mg/day
Vitamin D	7.5 μg/day
Calcium	1000 mg/day
Magnesium	350 mg/day
Iron	12.5 mg/day
Potassium	3000 mg/day

SFAs, saturated fatty acids; PUFAs, polyunsaturated fatty acids; MUFAs, monounsaturated fatty acids.

**Table 2:** Characteristics of the intervention cohort.

Characteristic	Juniors (*n* = 6)	Seniors (*n* = 8)	*p*-Value
Age (years)	14.3 ± 0.5	17.4 ± 1.4	0.002 *
Height (cm)	164.0 ± 4.8	167.0 ± 3.5	0.294
Body mass (kg)	51.7 ± 2.8	54.6 ± 4.1	0.170
Training (h/week)	10.3 ± 7.6	18.0 ± 5.7	0.051

* Mann–Whitney U test was used for age because normality was violated; other *p*-values are from independent-samples *t*-tests. Data are mean ± SD.

**Table 3:** Reported energy and macronutrient intake relative to fixed protocol benchmarks (exploratory comparisons).

Variable	Reference	Juniors Mean ± SD	*p* vs. Ref.	Seniors Mean ± SD	*p* vs. Ref.	*p* Between Groups
Energy (% requirement)	100	63.7 ± 18.2	0.004	67.1 ± 16.5	<0.001	0.718
Carbohydrate (g/kg/day)	5.5	3.91 ± 0.98	0.011	4.10 ± 1.24	0.015	0.768
Fat (% energy)	30	34.00 ± 6.06	0.167	35.80 ± 3.98	0.004	0.504
Protein (g/kg/day)	1.25	1.27 ± 0.43	0.911	1.68 ± 0.30	0.004	0.053
Fibre (g/day)	25	10.10 ± 4.25	<0.001	13.30 ± 5.59	<0.001	0.260
SFAs (% energy)	8	8.58 ± 3.01	0.656	6.07 ± 0.62	<0.001	0.039
PUFAs (% energy)	10	1.46 ± 1.45	0.031	2.08 ± 1.78	0.014	0.332 *
MUFAs (% energy)	12	2.46 ± 2.63	0.031	4.01 ± 1.32	0.014	0.045 *

Data are presented as mean ± SD. The reference values represent prespecified study-specific nutritional benchmarks rather than individual age-adjusted dietary requirements. The column “*p* vs. ref.” indicates comparisons of each participant’s mean intake over three recorded days against the corresponding fixed benchmark using one-sample *t*-tests or Wilcoxon signed-rank tests, as appropriate. Between-group comparisons were performed using independent-samples *t*-tests, with Welch’s correction when variances were unequal, or Mann–Whitney U tests (marked with *). All *p*-values are unadjusted and exploratory. Statistically significant differences from the benchmarks do not establish individual nutrient deficiency. SFAs, saturated fatty acids; PUFAs, polyunsaturated fatty acids; MUFAs, monounsaturated fatty acids.

**Table 4:** Reported micronutrient intake relative to fixed protocol benchmarks (exploratory comparisons).

Micronutrient	Reference	Juniors Mean ± SD	*p* vs. Ref.	Seniors Mean ± SD	*p* vs. Ref.	*p* Between Groups
Vitamin A (mg/day)	1	7.26 ± 7.76	0.105	5.84 ± 10.90	0.726	0.332 *
Folate (μg/day)	300	318 ± 310	0.890	174 ± 146	0.080	0.265
Vitamin B12 (μg/day)	1.75	0.94 ± 1.06	0.219	1.46 ± 0.70	0.284	0.279
Vitamin C (mg/day)	80	141 ± 131	0.301	60.0 ± 34.5	0.145	0.113
Vitamin D (μg/day)	7.5	0.72 ± 0.65	<0.001	1.83 ± 1.52	0.014	0.174 *
Calcium (mg/day)	1000	396 ± 197	<0.001	423 ± 183	<0.001	0.798
Magnesium (mg/day)	350	162.0 ± 85.6	0.003	165.0 ± 47.3	<0.001	0.925
Iron (mg/day)	12.5	7.55 ± 3.82	0.025	9.07 ± 4.23	0.141	0.561 *
Potassium (mg/day)	3000	1433 ± 333	<0.001	1619 ± 527	<0.001	0.464

Data are presented as mean ± SD. Reference values represent prespecified study-specific nutritional benchmarks and are not individually adjusted for age. The column “*p* vs. ref.” indicates one-sample *t*-tests or Wilcoxon signed-rank tests comparing each participant’s three-day mean intake with the corresponding benchmark. Between-group comparisons were performed using independent-samples *t*-tests, with Welch’s correction when variances were unequal, or Mann–Whitney U tests (marked with *). All *p*-values are unadjusted and exploratory. Differences from study benchmarks should not be interpreted as confirmed clinical nutrient deficiencies.

**Table 5:** Body composition at the baseline (M1) and approximately three months later (M2).

Variable	Juniors M1	Juniors M2	*p* (Effect)	Seniors M1	Seniors M2	*p* (Effect)
Body mass (kg)	51.7 ± 2.8	51.6 ± 3.5	0.862 (d = 0.075)	54.6 ± 4.1	54.8 ± 3.9	0.336 (d = −0.365)
Skeletal muscle mass (kg)	22.9 ± 1.2	22.9 ± 1.4	0.702 (d = −0.165)	25.3 ± 2.3	26.4 ± 4.7	0.798 (r = −0.143)
Fat mass (kg)	9.5 ± 1.2	9.4 ± 1.3	0.889 (d = 0.060)	8.6 ± 1.2	9.1 ± 1.2	0.119 (d = −0.628)
Body fat (%)	18.3 ± 1.6	18.2 ± 1.4	0.889 (d = 0.060)	15.7 ± 2.2	16.5 ± 2.1	0.119 (d = −0.628)
BMI (kg/m^2^)	19.3 ± 1.3	19.1 ± 1.4	0.479 (d = 0.312)	19.7 ± 1.4	19.8 ± 1.3	0.413 (d = −0.308)
ECW/TBW	0.383 ± 0.007	0.380 ± 0.004	0.055 (d = 1.015)	0.377 ± 0.005	0.378 ± 0.007	0.365 (d = −0.343)
Total body water (L)	30.9 ± 1.51	30.9 ± 1.8	0.674 (d = 0.182)	33.6 ± 2.8	33.4 ± 2.7	0.318 (d = 0.380)
Fat-free mass (kg)	42.3 ± 2.1	42.2 ± 2.4	0.862 (d = 0.075)	46.0 ± 3.8	45.7 ± 3.6	0.283 (d = 0.411)

Data are presented as mean ± SD. Within-group comparisons between baseline (M1) and follow-up (M2) were performed using paired-samples *t*-tests or Wilcoxon signed-rank tests when normality assumptions were not satisfied. For paired comparisons, effect sizes were calculated using the M1 − M2 contrast; positive values therefore generally indicate decreases between assessments, whereas negative values indicate increases. Cohen’s d was reported for parametric comparisons, and rank-biserial correlation (r) for non-parametric comparisons. The sign of an effect size does not imply clinical improvement or deterioration. BMI, body mass index; ECW/TBW, extracellular-to-total body water ratio.

**Table 6:** Comparison of body-composition change scores between junior and senior gymnasts.

Variable	Juniors Δ	Seniors Δ	*p*-Value	Effect Size
Body mass (kg)	−0.067	0.225	0.481	d = −0.392
Skeletal muscle mass (kg)	0.067	1.063	0.845	r = −0.083
Fat mass (kg)	−0.017	0.463	0.224	d = −0.692
Body fat (%)	−0.067	0.800	0.210	d = −0.716
BMI (kg/m^2^)	−0.150	0.075	0.272	d = −0.622
ECW/TBW	−0.00350	0.00075	0.015	d = −1.527
Total body water (L)	−0.083	−0.238	0.621	d = 0.274
Fat-free mass (kg)	−0.050	−0.325	0.506	d = 0.370

Individual change scores were calculated as Δ = M2 − M1. Between-group comparisons were performed using independent-samples *t*-tests, Welch’s *t*-tests when variances were unequal, or Mann–Whitney U tests, as appropriate. Cohen’s d and rank-biserial correlation (r) were used for parametric and non-parametric comparisons, respectively. Effect-size signs reflect the junior-minus-senior contrast of change scores and should not be interpreted as indicators of clinical benefit or harm. All *p*-values are unadjusted and exploratory. BMI, body mass index; ECW/TBW, extracellular-to-total body water ratio.

## Data Availability

The raw data supporting the conclusions of this article will be made available by the authors on request. Due to the nature of the data and to ensure responsible use and proper interpretation, the datasets are not publicly available. The main raw data were provided during the submission.

## Abbreviations

The following abbreviations are used in this manuscript:

BMR	Basal metabolic rate
BMI	Body mass index
ECW/TBW	Extracellular water-to-total body water ratio
IOC	International Olympic Committee
LEA	Low energy availability
MUFAs	Monounsaturated fatty acids
PAL	Physical activity level
PUFAs	Polyunsaturated fatty acids
RED-S	Relative energy deficiency in sport
SFAs	Saturated fatty acids
